# Parameterized Multipartite Entanglement and Genuine Entanglement Measures Based on *q*-Concurrence

**DOI:** 10.3390/e26070535

**Published:** 2024-06-22

**Authors:** Pan-Wen Ma, Hui Zhao, Shao-Ming Fei, Mei-Ming Zhang, Zhi-Xi Wang

**Affiliations:** 1 School of Mathematics, Statistics and Mechanics, Beijing University of Technology, Beijing 100124, China; 2 School of Mathematical Sciences, Capital Normal University, Beijing 100048, China; 3 School of Mathematics and Physics, Jiangsu University of Technology, Changzhou 213001, China

**Keywords:** genuine multipartite entanglement measure, multipartite entanglement measure, *q*-concurrence

## Abstract

We study genuine multipartite entanglement (GME) and multipartite *k*-entanglement based on *q*-concurrence. Well-defined parameterized GME measures and measures of multipartite *k*-entanglement are presented for arbitrary dimensional *n*-partite quantum systems. Our GME measures show that the GHZ state is more entangled than the *W* state. Moreover, our measures are shown to be inequivalent to the existing measures according to entanglement ordering. Detailed examples show that our measures characterize the multipartite entanglement finer than some existing measures, in the sense that our measures identify the difference of two different states while the latter fail.

## 1. Introduction

Quantum entanglement serves as a valuable physical resource in quantum information processing, enabling tasks beyond classical ones [1]. It plays a crucial role in various quantum information tasks such as quantum computing [2,3,4], dense coding [5], and quantum teleportation [6,7,8].

Several methods have been developed for quantifying the entanglement of quantum states. For multipartite quantum system, Ma et al. introduced a genuine multipartite entanglement measure, known as the genuine multipartite concurrence (GMC), in which the entanglement of any pure state is quantified by selecting the minimum bipartite concurrence over all possible bipartite splits [9]. A new GME measure, the geometric mean of bipartite concurrence (GBC), was introduced in [10]. Although it does not involve the minimization in calculating the entanglement of pure states, it can only distinguish between genuine entangled states and nongenuine entangled states. For bipartite states, a series of entanglement measures have been proposed, such as entanglement distillation [11,12], entanglement formation [13], negativity [14], and concurrence [15,16]. In [17], Hong et al. proposed a measure of entanglement called *k* (2≤k≤n) multipartite entanglement (*k*-ME) concurrence of any *n*-partite states, which satisfies the key properties of a well-defined measure of entanglement, such as strictly greater than zero for all *k*-nonseparable states, vanishing on *k*-separable states, invariance under local unitary transformations and convexity. Moreover, it satisfies the entanglement monotonicity, namely, the entanglement does not increase under local operations and classical communication (LOCC). Recently, a class of entanglement measures called *k* geometric mean (*k*-GM) concurrence has been presented based on the geometric mean of the entanglement associated with *k*-partitions of *n*-partite quantum systems [18]. Inspired by general Tsallis entropy [19], by using *q*-concurrence, the authors in [20] provided the parameterized entanglement measures as the generalizations of the *k*-ME concurrence.

Given that the multipartite entanglement plays a significant role in quantum information processing, the characterization and quantification of multipartite entanglement have been extensively investigated. Nevertheless, due to the extremely complex structure of entanglement in multipartite quantum states, the studies on GME and multipartite *k*-entanglement are still far from being satisfied. By taking into account that the *q*-concurrence is nonincreasing under local operations and classical communication, and vanishes for biseparable states, we present both GME measures and *k*-entanglement measures based on *q*-concurrence.

In this paper, we construct GME measures and *k*-entanglement measures for multipartite quantum systems in terms of *q*-concurrence. The paper is organized as follows. In Section 2, we review some fundamental concepts and give well-defined parameterized GME measures for multipartite quantum systems. By detailed examples, we show that our measures are more efficient than the existing ones in detecting the genuine multipartite entanglement. In Section 3, we present parameterized *k*-entanglement measures for arbitrary dimensional *n*-partite systems by using *q*-concurrence. Through detailed examples, we demonstrate that our measures have different state ordering from other ones. Conclusions are given in Section 4.

## 2. Parameterized GME Measures for Multipartite Pure States

We first focus on genuine multipartite entanglement. A multipartite pure state is genuinely multipartite entangled if it is not biseparable with respect to any bipartition. A well-defined GME measure should satisfy the following conditions: (a) For all product and biseparable states, the measure must be zero. (b) It is strictly positive for all non-biseparable states. (c) It is nonincreasing under local operations and classical communications.

Denote $H_{f}$ as a $d_{f}$-dimensional Hilbert vector space. The concurrence of a bipartite pure state $|\psi_{12}\rangle \in H_{1}⊗H_{2}$ is given by $C(|\psi_{12}\rangle )=\sqrt{2(1-Tr\left(\rho_{1}^{2}\right))}$, where $\rho_{1}=Tr_{2}(|\psi_{12}\rangle \langle \psi_{12}|)$. The *q*-concurrence (q≥2) is defined by [19], $C_{q}(|\psi_{12}\rangle )=1-Tr\left(\rho_{1}^{q}\right)$. For arbitrary *n*-partite pure state $|\psi \rangle \in H_{1}⊗H_{2}⊗\cdots ⊗H_{n}$, the *q*-concurrence under bipartition $J_{t}|\hat{J_{t}}$ is given by

(1) $$C_{qJ_{t}|\hat{J_{t}}}(|\psi \rangle )=1-Tr\left(\rho_{J_{t}}^{q}\right),$$

where $J_{t}$$\left(t=1,2,\cdots ,2^{n-1}-1\right)$ is a subsystem of $H_{1}⊗H_{2}⊗\cdots ⊗H_{n}$ and $\hat{J_{t}}$ is the complement of $J_{t}$. Conditions (a) and (b) of a well-defined GME measure imply that one needs to take over all possible bipartitions in constructing GME measures. Hence, we choose the form of geometric mean of concurrence. Moreover, we add a parameter to ensure that our constructed GME measures satisfy condition (c) for well-defined GME measures. In terms of the *q*-concurrence, we have the following parameterized GME measures.

**Theorem** **1.** *For any n-partite pure states $|\psi \rangle \in H_{1}⊗H_{2}⊗\cdots ⊗H_{n}$,*

(2) $$\Gamma_{GME}\left(|\psi \rangle \right){=\left[\prod_{J_{t}}C_{qJ_{t}|\hat{J_{t}}}^{\xi }(|\psi \rangle \right)]}^{\frac{1}{2^{n-1}-1}}$$

*is a well-defined GME measure for 0<ξ≤1.*

**Proof.**  We first prove that |ψ〉 is genuine entangled if and only if $\Gamma_{GME}(|\psi \rangle )>0$. Consider the continuous function $y=x^{\xi }$(0<ξ≤1). If y>0, then $x=y^{\frac{1}{\xi }}>0$. Hence, if $\Gamma_{GME}(|\psi \rangle )>0$, then $C_{qJ_{t}|\hat{J_{t}}}^{\xi }(|\psi \rangle )>0$, and $C_{qJ_{t}|\hat{J_{t}}}(|\psi \rangle )>0$ as well. That is to say, $C_{qJ_{t}|\hat{J_{t}}}(|\psi \rangle )>0$ always holds under any bipartition. Hence, |ψ〉 is a genuine multipartite entangled state. On the other hand, if $\Gamma_{GME}(|\psi \rangle )=0$, then there must exist $C_{qJ_{t}|\hat{J_{t}}}(|\psi \rangle )=0$ under certain bipartition $J_{t}|\hat{J_{t}}$, namely, |ψ〉 is not a genuine multipartite entangled state.

We next prove that $\Gamma_{GME}(|\psi \rangle )$ does not increase under LOCC. As the *q*-concurrence does not increase under LOCC [20], we only need to verify that $\Gamma_{GME}(|\psi \rangle )$ is an increasing function of $C_{qJ_{m}|\hat{J_{m}}}(|\psi \rangle )$. In fact,

(3) $$\frac{\partial \Gamma_{GME}(|\psi \rangle )}{\partial C_{qJ_{m}|\hat{J_{m}}}(|\psi \rangle )}=\frac{\xi \left[C_{qJ_{m}|\hat{J_{m}}}(|\psi \rangle \right){]}^{\frac{\xi }{2^{n-1}-1}-1}}{2^{n-1}-1}\left[\prod_{t\neq m}C_{qJ_{t}|\hat{J_{t}}}^{\xi }(|\psi \rangle \right){]}^{\frac{1}{2^{n-1}-1}}\geq 0$$

for $m\in \{1,2,\cdots ,2^{n-1}-1\}$. Thus, the monotonicity of $\Gamma_{GME}(|\psi \rangle )$ holds, and $\Gamma_{GME}(|\psi \rangle )$ is nonincreasing under LOCC. Therefore, $\Gamma_{GME}(|\psi \rangle )$ is a bona fide measure of GME. □

In [21], the authors suggested that a proper GME measure should satisfy an additional criterion: (d) the GME of the GHZ state is larger than that of the *W* state. Here, for four-qubit pure GHZ state $|GHZ\rangle =\frac{1}{\sqrt{2}}(|0000\rangle +\left|1111\rangle \right)$ and the *W* state $|W\rangle =\frac{1}{2}(|1000\rangle +|0100\rangle +|0010\rangle +\left|0001\rangle \right)$, we obtain for q=2 and ξ=1, $\Gamma_{GME}(|GHZ\rangle )=1$ due to $C_{qJ_{t}|\hat{J_{t}}}(|\psi \rangle )$ being all equal to 1, while $\Gamma_{GME}(|W\rangle )=0.4242$. Obviously, the GHZ state is more entangled than the *W* state. Thus, $\Gamma_{GME}(|\psi \rangle )$ is also a proper GME measure in this sense.

**Example** **1.** *Let us consider the following four-qubit pure states,*

$$\begin{array}{cc} |\psi_{A}\rangle & =\frac{1}{2}\left(|0000\rangle +|1011\rangle +|1101\rangle +|1110\rangle \right),\\ |\psi_{B}\rangle & =\frac{1}{2}\left(|0000\rangle +|0101\rangle +|1000\rangle +|1110\rangle \right),\\ |\psi_{C}\rangle & =\frac{1}{\sqrt{5}}\left(|0000\rangle +|1111\rangle +|0011\rangle +|0101\rangle +|0110\rangle \right),\\ |\psi_{D}\rangle & =\frac{1}{\sqrt{4(\frac{5\sqrt{113}}{32}+\frac{51}{32})+3}}(\sqrt{4(\frac{5\sqrt{113}}{32}+\frac{51}{32})}(|0000\rangle +|0101\rangle +|1010\rangle +\left|1111\rangle \right)\\ \; & +(i|0001\rangle +|0110\rangle -i|1011\rangle )), \end{array}$$

*where $i=\sqrt{-1}$.*

The genuine multipartite concurrence of a four-qubit quantum pure state |ψ〉 is defined to be $GMC(|\psi \rangle )=\min_{\gamma_{t}\in \gamma }\sqrt{2(1-Tr{\left(\rho_{\gamma_{t}}\right)}^{2})}$ in [9], where $\gamma =\{\gamma_{t}\}$ labels all the different reduced density matrices of |ψ〉〈ψ|. Direct calculation shows that $GMC(|\psi_{A}\rangle )=GMC(|\psi_{B}\rangle )=0.8660$ and $GMC(|\psi_{C}\rangle )=GMC(|\psi_{D}\rangle )=0.8000$. It is evident that although the GMC detects genuine multipartite entanglement of above four-qubit pure states, it cannot tell the difference in entanglement neither between $|\psi_{A}\rangle$ and $|\psi_{B}\rangle$ nor between $|\psi_{C}\rangle$ and $|\psi_{D}\rangle$. The fact is due to GMC only depending on the minimum of concurrence, which is the same for both $|\psi_{A}\rangle$ and $|\psi_{B}\rangle$ as well as for both $|\psi_{C}\rangle$ and $|\psi_{D}\rangle$.

From our Theorem 1, we have $\Gamma_{GME}(|\psi_{A}\rangle )=0.8083$, $\Gamma_{GME}(|\psi_{B}\rangle )=0.7699$, $\Gamma_{GME}(|\psi_{C}\rangle )$=0.1133 and $\Gamma_{GME}(|\psi_{D}\rangle )=0.8593$ for q=2 and $\xi =\frac{1}{3}$. Therefore, $|\psi_{A}\rangle$ (or $|\psi_{D}\rangle$) is more entangled than $|\psi_{B}\rangle$ (or $|\psi_{C}\rangle$). Namely, our measure $\Gamma_{GME}(|\psi \rangle )$ can distinguish the difference in the entanglement between $|\psi_{A}\rangle$ and $|\psi_{B}\rangle$ as well as between $|\psi_{C}\rangle$ and $|\psi_{D}\rangle$. In this sense, our measure characterizes the genuine multipartite entanglement in a more fine way.

Furthermore, concerning the entanglement order [21,22], any two entanglement measures should give rise to the same ordering on the set of entangled states if they are actually equivalent [23]. Namely, if two entanglement measures $E_{1}$ and $E_{2}$ are equivalent, then $E_{1}\left(\rho_{1}\right)\geq E_{1}\left(\rho_{2}\right)$ implies $E_{2}\left(\rho_{1}\right)\geq E_{2}\left(\rho_{2}\right)$ for any pair of $\rho_{1}$ and $\rho_{2}$. The following example shows that two entanglement measures are inequivalent when there exists a different entanglement order in certain intervals. We will highlight the advantages of our measure by comparing it with other measures.

**Example** **2.** *Consider the following family of four-qubit pure states,*

(4) $$\begin{array}{c} |\phi_{\theta }\rangle =\sin\theta (\cos\left(\frac{2\pi }{3}\right)|0100\rangle +\sin\left(\frac{2\pi }{3}\right)\left|1000\rangle \right)+\cos\theta |0011\rangle , \end{array}$$

**Figure 1:**
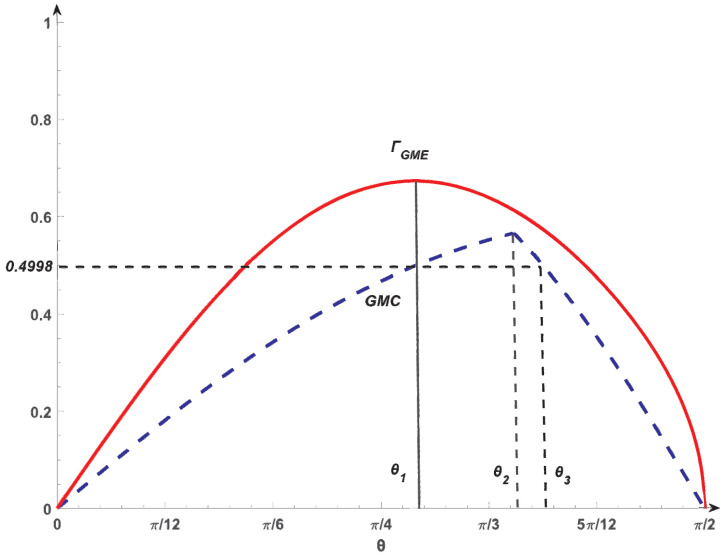
The $\Gamma_{GME}$ (solid red) and GMC (dashed blue) for the four-qubit states given in (4) versus θ. The peak of $\Gamma_{GME}$ is at $\theta_{1}$, while the GMC has a sharp peak at $\theta =\theta_{2}$. From $\theta_{1}$ to $\theta_{2}$, $\Gamma_{GME}$ decreases, while the GMC increases. The GMC has a series of paired equal values in the interval $\theta \in [\theta_{1},\theta_{3}]$.

*with $\theta \in [0,\frac{\pi }{2}]$. Set $\xi =\frac{1}{2}$ and q=2. Using Theorem 1, we have that $|\phi_{\theta }\rangle$ is genuine entangled for $\theta \in (0,\frac{\pi }{2})$, see Figure 1. With the increasing in θ from $\theta_{1}$ to $\theta_{2}$, $\Gamma_{GME}$ decreases. Nevertheless, the GMC increases from $\theta_{1}$ to $\theta_{2}$ [9]. Thus, for any two arbitrary states within this range, the entanglement order for GMC and $\Gamma_{GME}$ is different. GMC and $\Gamma_{GME}$ are inequivalent in this sense. Meanwhile, $\Gamma_{GME}$ is a smooth function of θ, while GMC displays a sharp peak at $\theta_{2}=1.1071$.*

Moreover, for $\theta_{1}=0.8711$ and $\theta_{3}=1.1783$, one has $GMC(|\phi_{\theta_{1}}\rangle )=GMC(|\phi_{\theta_{3}}\rangle )=0.4998$ by using the GMC in [9]. This is due to the fact that GMC increases from $\theta_{1}$ to $\theta_{2}$ and decreases from $\theta_{2}$ to $\theta_{3}$. Meanwhile, from our GME measure, we have $\Gamma_{GME}\left(|\phi_{\theta_{1}}\rangle \right)=0.6731$ and $\Gamma_{GME}\left(|\phi_{\theta_{3}}\rangle \right)=0.5733$, as $\Gamma_{GME}$ decreases from $\theta_{1}$ to $\theta_{3}$. This means that $|\phi_{\theta_{1}}\rangle$ is more entangled than $|\phi_{\theta_{3}}\rangle$ from our GME measure, while the GMC fails to detect this difference. In this sense, our GME measure shows superior performance in characterizing the genuine multipartite entanglement.

## 3. Parameterized *k*-Entanglement Measures for *n*-Partite Quantum Systems

An *n*-partite pure state |ψ〉 is separable under *k*-partition if it can be expressed as $\left|\psi \rangle =\right|\psi_{1}\rangle_{J_{1}}⊗|\psi_{2}\rangle_{J_{2}}⊗\cdots ⊗{|\psi_{k}\rangle }_{J_{k}}$, where $|\psi_{l}\rangle_{J_{l}}$ is the state in subsystem $J_{l}$ of the *k*-partition $J_{1}|J_{2}\left|\cdots \right|J_{k}$ in the set S={1,2,⋯,n}. This *k*-partition strictly obeys the following conditions: (i) $\overset{k}{\underset{l=1}{⋃}}J_{l}=S$; (ii) $J_{u}⋂J_{v}=\emptyset$ when u≠v. Similarly, an *n*-partite mixed state ρ is *k*-separable if it can be represented as a convex mixture of *k*-separable pure states, i.e., $\rho =\sum_{i}p_{i}|\psi_{i}\rangle \langle \psi_{i}|$, where $\left\{|\psi_{i}\rangle \right\}$ is *k*-separable with respect to certain *k*-partitions. Otherwise, ρ is *k*-nonseparable.

We denote the set of all *k*-separable states by $S_{k}$(k=2,3,⋯,n), with $S_{1}$ denoting the set of all quantum states. Clearly, $S_{n}\subset S_{n-1}\subset \cdots \subset S_{1}$. In particular, the complement $S_{1}\setminus S_{2}$ is the set containing all genuine multipartite entangled (2-nonseparable) states.

An entanglement measure for *k*-separability has to satisfy the following conditions: (i) For all *k*-separable states, the measure must be zero. (ii) For all *k*-nonseparable states, the measure must be positive. (iii) It is invariant under local unitary transformations. (iv) The measure is nonincreasing under LOCC for any state ρ (monotonicity). (v) The measure never increases under free operations of LOCC for its LOCC-ensemble $\left\{p_{j},\sigma_{j}\right\}$ (strong monotonicity). (vi) Convexity $\left(E\left(\sum_{i}p_{i}\rho_{i}\right)\leq \sum_{i}p_{i}E\left(\rho_{i}\right)\right)$. Here, the monotonicity means that the measure does not increase under any LOCC, i.e., $E(\Lambda_{\mathrm{LOCC}}\left(\rho \right))\leq E\left(\rho \right)$. The strong monotonicity says that if ρ is transformed into a state $\sigma_{j}$ with the probability $p_{j}$ under LOCC, the measure is still nonincreasing on average, namely, $\sum_{j}p_{j}E\left(\sigma_{j}\right)\leq E\left(\rho \right)$ holds for the LOCC-ensemble $\left\{p_{j},\sigma_{j}\right\}$.

According to the above conditions (i) and (ii) for a well-defined measure of *k*-entanglement, we need to take into account all possible *k*-partitions of multipartite states. By using the concavity of the function $y=x^{\xi }$(0<ξ≤1) and the fact that the function $g={\left[\prod_{i=1}^{n}x_{i}\right]}^{\frac{1}{n}}$ is concave [24], we construct *k*-entanglement measures satisfying conditions (iii)–(vi) by adding some parameters. To quantify the *k*-entanglement with respect to the *k*-separability of *n*-partite systems, we first present parameterized *k*-entanglement measures k=2,3,⋯,n for any *n*-partite pure states |ψ〉,

(5) $$\Gamma_{k-ME}(|\psi \rangle )=[\prod_{\alpha_{i}\in L_{k}}(\frac{\sum_{t=1}^{k}C_{qJ_{t\alpha_{i}}|{\hat{J}}_{t\alpha_{i}}}^{\xi }(|\psi \rangle )}{k}{)}^{\frac{1}{r}}{]}^{\frac{1}{|L_{k}|}},\;0<\xi \leq 1,r\geq 1,$$

where

(6) $$|L_{k}|=\sum_{t=1}^{k}\frac{{\left(-1\right)}^{k-t}t^{n-1}}{(t-1)!(k-t)!},$$

where $J_{t\alpha_{i}}|{\hat{J}}_{t\alpha_{i}}$ represents any bipartition of the state |ψ〉, $L_{k}=\left\{\alpha_{i}\right\}$ stands for the set that encompasses all possible *k*-partitions $\left\{J_{1\alpha_{i}}|J_{2\alpha_{i}}|\cdots |J_{k\alpha_{i}}\right\}$, and $|L_{k}|$ denotes the cardinality of the elements in the set $L_{k}$. $\Gamma_{k-ME}(|\psi \rangle )$ is generalized to *n*-partite mixed states by convex-roof extension,

(7) $$\Gamma_{k-ME}\left(\rho \right)=\underset{\left\{p_{i},|\psi_{i}\rangle \right\}}{\inf}\sum_{i}p_{i}\Gamma_{k-ME}(|\psi_{i}\rangle ),$$

where the infimum is taken over all possible pure state decompositions. We have the following conclusion.

**Theorem** **2.** *For any n-partite mixed state ρ, $\Gamma_{k-ME}\left(\rho \right)$ are well-defined parameterized measures of k-entanglement of n-partite systems.*

**Proof.**  (i) $\Gamma_{k-ME}\left(\rho \right)=0$ for any $\rho \in S_{k}$ (vanishing on all *k*-separable states).

If a pure state |ψ〉 is *k*-separable, then there must exist $C_{qJ_{t\alpha_{i}}|\hat{J}t\alpha_{i}}(|\psi \rangle )$ such that $\Gamma_{k-ME}(|\psi \rangle )=0$ under certain *k*-partitions. Likewise, for an arbitrary mixed *k*-separable state ρ with pure state decomposition $\left\{p_{i},|\psi_{i}\rangle \right\}$, we have $\Gamma_{k-ME}\left(\rho \right)\leq \sum_{i}p_{i}\Gamma_{k-ME}(|\psi_{i}\rangle )=0$.

(ii) $\Gamma_{k-ME}\left(\rho \right)>0$ for all *k*-nonseparable states ρ.

If an arbitrary pure state |ψ〉 is *k*-nonseparable, there always exists $C_{qJ_{t\alpha_{i}}|\hat{J}t\alpha_{i}}(|\psi \rangle )>0$ under *k*-partition such that $\Gamma_{k-ME}(|\psi \rangle )>0$. Then, if ρ is a mixed *k*-nonseparable state, it cannot be expressed as a convex mixture of *k*-separable pure states, i.e., $\Gamma_{k-ME}\left(\rho \right)>0$.

(iii) $\Gamma_{k-ME}\left(U_{Local}^{†}\rho U_{Local}\right)=\Gamma_{k-ME}\left(\rho \right)$ (invariant under local unitary transformations).

Since the *q*-concurrence is invariant under local unitary transformations, we have

(8) $$\begin{array}{cc} \Gamma_{k-ME}\left(U_{Local}^{†}\rho U_{Local}\right)\; & =[\prod_{\alpha_{i}\in L_{k}}(\frac{\sum_{t=1}^{k}C_{qJ_{t\alpha_{i}}|{\hat{J}}_{t\alpha_{i}}}^{\xi }\left(U_{Local}^{†}\rho U_{Local}\right)}{k}{)}^{\frac{1}{r}}{]}^{\frac{1}{|L_{k}|}}\\ \; & =[\prod_{\alpha_{i}\in L_{k}}(\frac{\sum_{t=1}^{k}C_{qJ_{t\alpha_{i}}|{\hat{J}}_{t\alpha_{i}}}^{\xi }\left(\rho \right)}{k}{)}^{\frac{1}{r}}{]}^{\frac{1}{|L_{k}|}}\\ \; & =\Gamma_{k-ME}\left(\rho \right). \end{array}$$

(iv) $\Gamma_{k-ME}\left(\rho \right)$ is nonincreasing under LOCC (monotonicity).

Firstly, we prove that the inequality $\Gamma_{k-ME}\left(\Lambda_{\mathrm{LOCC}}(|\psi \rangle \right))\leq \Gamma_{k-ME}(|\psi \rangle )$ holds for any LOCC operations on pure state |ψ〉. Since the *q*-concurrence decreases under LOCC, we only need to verify that $\Gamma_{k-ME}(|\psi \rangle )$ is an increasing function of $C_{qJ_{m\alpha_{l}}|{\hat{J}}_{m\alpha_{l}}}(|\psi \rangle )$. By direct calculation, we have

(9) $$\begin{array}{c} \frac{\partial \Gamma_{k-ME}(|\psi \rangle )}{\partial C_{qJ_{m\alpha_{l}}|{\hat{J}}_{m\alpha_{l}}}(|\psi \rangle )}=\frac{\xi C_{qJ_{m\alpha_{l}}|{\hat{J}}_{m\alpha_{l}}}^{\xi -1}\left(|\psi \rangle \right)\prod_{L_{k}/\left\{\alpha_{l}\right\}}\sum_{t=1}^{k}C_{qJ_{t\alpha_{l}}|{\hat{J}}_{t\alpha_{l}}}^{\xi }\left(|\psi \rangle \right)}{rk|L_{k}{\left|\left[\prod_{\alpha_{i}\in L_{k}}\sum_{t=1}^{k}C_{qJ_{t\alpha_{i}}|{\hat{J}}_{t\alpha_{i}}}^{\xi }(|\psi \rangle \right)\right]}^{1-\frac{1}{r|L_{k}|}}}\geq 0, \end{array}$$

where $l=1,2,\cdots ,|L_{k}|$, m=1,2,⋯,k. Hence, $\Gamma_{k-ME}(|\psi \rangle )$ is nonincreasing under LOCC.

Now, for an arbitrary mixed state ρ with pure state decomposition $\left\{p_{i},|\psi_{i}\rangle \right\}$, we obtain

(10) $$\begin{array}{cc} \Gamma_{k-ME}\left(\Lambda_{LOCC}\left(\rho \right)\right)\; & =\Gamma_{k-ME}\left(\Lambda_{LOCC}(\sum_{i}p_{i}|\psi_{i}\rangle \langle \psi_{i}|)\right)\\ \; & =\Gamma_{k-ME}\left(\sum_{i}p_{i}\Lambda_{LOCC}(|\psi_{i}\rangle \langle \psi_{i}|)\right)\\ \; & \leq \sum_{i}p_{i}\Gamma_{k-ME}\left(\Lambda_{LOCC}(|\psi_{i}\rangle \right))\\ \; & \leq \sum_{i}p_{i}\Gamma_{k-ME}(|\psi_{i}\rangle )\\ \; & =\Gamma_{k-ME}\left(\rho \right), \end{array}$$

where the first inequality is due to the convexity of parameterized *k*-entanglement measures $\Gamma_{k-ME}\left(\rho \right)$, and the second inequality is derived from the property that $\Gamma_{k-ME}(|\psi \rangle )$ is nonincreasing under LOCC for any pure states.

(v) $\Gamma_{k-ME}\left(\rho \right)$ never increases under free operations of LOCC for its LOCC-ensemble $\left\{p_{j},\sigma_{j}\right\}$ (strong monotonicity).

We need to prove that the inequality $\Gamma_{k-ME}\left(\rho \right)\geq \sum_{j}p_{j}\Gamma_{k-ME}\left(\sigma_{j}\right)$ holds, where the state $\sigma_{j}=K_{j}\left|\psi \rangle \langle \psi \right|K_{j}^{†}$ is generated with probability $p_{j}$ by applying LOCC on ρ, $\sum_{j}K_{j}^{†}K_{j}=I$ (unit operator). If ρ=|ψ〉〈ψ| is a pure state, we have

(11) $$\begin{array}{cc} \Gamma_{k-ME}\left(\rho \right)\; & =[\prod_{\alpha_{i}\in L_{k}}(\frac{\sum_{t=1}^{k}C_{qJ_{t\alpha_{i}}|{\hat{J}}_{t\alpha_{i}}}^{\xi }\left(\rho \right)}{k}{)}^{\frac{1}{r}}{]}^{\frac{1}{|L_{k}|}}\\ \; & \geq [\prod_{\alpha_{i}\in L_{k}}(\frac{\sum_{t=1}^{k}{\left(\sum_{j}p_{j}C_{qJ_{t\alpha_{i}}|{\hat{J}}_{t\alpha_{i}}}\left(\sigma_{j}\right)\right)}^{\xi }}{k}{)}^{\frac{1}{r}}{]}^{\frac{1}{|L_{k}|}}\\ \; & \geq [\prod_{\alpha_{i}\in L_{k}}(\frac{\sum_{t=1}^{k}\sum_{j}p_{j}C_{qJ_{t\alpha_{i}}|{\hat{J}}_{t\alpha_{i}}}^{\xi }\left(\sigma_{j}\right)}{k}{)}^{\frac{1}{r}}{]}^{\frac{1}{|L_{k}|}}\\ \; & =[\prod_{\alpha_{i}\in L_{k}}(\frac{\sum_{j}p_{j}\sum_{t=1}^{k}C_{qJ_{t\alpha_{i}}|{\hat{J}}_{t\alpha_{i}}}^{\xi }\left(\sigma_{j}\right)}{k}{)}^{\frac{1}{r}}{]}^{\frac{1}{|L_{k}|}}\\ \; & \geq \sum_{j}p_{j}[\prod_{\alpha_{i}\in L_{k}}(\frac{\sum_{t=1}^{k}C_{qJ_{t\alpha_{i}}|{\hat{J}}_{t\alpha_{i}}}^{\xi }\left(\sigma_{j}\right)}{k}{)}^{\frac{1}{r}}{]}^{\frac{1}{|L_{k}|}}\\ \; & \geq \sum_{j}p_{j}\Gamma_{k-ME}\left(\sigma_{j}\right), \end{array}$$

where the first inequality is due to the strong monotonicity of *q*-concurrence, that is, $C_{q}\left(\rho \right)\geq \sum_{j}p_{j}C_{q}\left(\sigma_{j}\right)$ [19]; the concavity of the function $y=x^{\xi }$(0<ξ≤1) leads to the second inequality; and the third inequality holds, as the function $g={\left[\prod_{i=1}^{n}x_{i}\right]}^{\frac{1}{n}}$ is concave [24].

For mixed state $\rho =\sum_{i}p_{i}|\psi_{i}\rangle \langle \psi_{i}|$, we have

(12) $$\begin{array}{cc} \Gamma_{k-ME}\left(\rho \right)\; & =\sum_{i}p_{i}\Gamma_{k-ME}\left(|\psi_{i}\rangle \right)\\ \; & \geq \sum_{ij}p_{i}Tr(K_{j}|\psi_{i}\rangle \langle \psi_{i}|K_{j}^{†})\Gamma_{k-ME}\left(\frac{K_{j}|\psi_{i}\rangle \langle \psi_{i}|K_{j}^{†}}{Tr(K_{j}|\psi_{i}\rangle \langle \psi_{i}|K_{j}^{†})}\right)\\ \; & =\sum_{ij}Tr\left(K_{j}\rho K_{j}^{†}\right)\frac{p_{i}Tr(K_{j}|\psi_{i}\rangle \langle \psi_{i}|K_{j}^{†})}{Tr(K_{j}\rho K_{j}^{†})}\Gamma_{k-ME}\left(\frac{K_{j}|\psi_{i}\rangle \langle \psi_{i}|K_{j}^{†}}{Tr(K_{j}|\psi_{i}\rangle \langle \psi_{i}|K_{j}^{†})}\right)\\ \; & =\sum_{j}p_{j}\left[\sum_{i}p_{ij}\Gamma_{k-ME}(|\psi_{ij}\rangle \right)]\\ \; & \geq \sum_{j}p_{j}\Gamma_{k-ME}\left(\sigma_{j}\right), \end{array}$$

where $|\psi_{ij}\rangle =\frac{K_{j}|\psi_{i}\rangle }{\sqrt{Tr(K_{j}|\psi_{i}\rangle \langle \psi_{i}|K_{j}^{†})}}$, $p_{ij}=\frac{p_{i}Tr(K_{j}|\psi_{i}\rangle \langle \psi_{i}|K_{j}^{†})}{Tr(K_{j}\rho K_{j}^{†})}$ and the state $\sigma_{j}=\sum_{i}p_{ij}|\psi_{ij}\rangle \langle \psi_{ij}|$ occurs with the probability $p_{j}=Tr\left(K_{j}\rho K_{j}^{†}\right)$ under LOCC. The first inequality holds since $\Gamma_{k-ME}(|\psi \rangle )$ obeys the strong monotonicity for any pure states. The second inequality is due to the definition of $\Gamma_{k-ME}\left(\rho \right)$.

(vi) Convexity $E\left(\sum_{i}p_{i}\rho_{i}\right)\leq \sum_{i}p_{i}E\left(\rho_{i}\right)$ is due to the convexity of the mixed states. □

In [17], the *k*-ME concurrence of an *n*-partite pure state |ψ〉 is defined by $C_{k-ME}(|\psi \rangle )=\min_{A}\sqrt{\frac{2\sum_{t=1}^{k}\left(1-Tr{\left(\rho_{A_{t}}\right)}^{2}\right)}{k}}$, where $\rho_{A_{t}}$ is the reduced density matrix of the subsystem $A_{t}$ and the minimum is taken over all possible *k*-partitions $A=A_{1}|A_{2}\left|\cdots \right|A_{k}$ of the set S={1,2,⋯,n}.

In [18], the *k*-GM concurrence of an *n*-partite pure state |ψ〉 is defined by $C_{k-GM}(|\psi \rangle )=[\prod_{\alpha_{i}\in T_{k}}\sqrt{\frac{2\sum_{t=1}^{k}C_{2A_{t\alpha_{i}}|{\hat{A}}_{t\alpha_{i}}}(|\psi \rangle )}{k}}{]}^{\frac{1}{|T_{k}|}}$, where $T_{k}=\left\{\alpha_{i}\right\}$ is the set of all possible *k*-partitions $A_{1\alpha_{i}}\left|A_{2\alpha_{i}}\right|\cdots$ where $|A_{k\alpha_{i}}$, $|T_{k}|$ represents the cardinality of the elements in the set $T_{k}$, and $C_{2}$ is the *q*-concurrence with q=2.

The example below illustrates that our approach is able to detect multipartite entanglement and is inequivalent to the above multipartite entanglement measures.

**Example** **3.** *Consider the following family of four-qubit pure states*

(13) $$|\psi_{\theta }\rangle =\frac{\sqrt{3}}{3}\sin\theta (|0001\rangle +|0100\rangle +\left|1000\rangle \right)+\cos\theta |0011\rangle ,$$

*with θ∈[0,π]. By using Equation (5) for $\xi =\frac{1}{2}$, r=1 and q=2, $|\psi_{\theta }\rangle$ is 3-nonseparable when θ∈(0,π). By direct calculation, 3-GM concurrence has a maximum value 0.9358 at $\theta_{1}=1.011$ in the interval $\left(0,\frac{\pi }{2}\right)$, while in the interval $\left(\frac{\pi }{2},\pi \right)$, the 3-GM concurrence has a maximum value 0.9358 at $\theta_{6}=2.1306$. The 3-ME concurrence takes a maximum value 0.9129 at $\theta_{3}=1.0472$ or $\theta_{4}=2.0944$. Our $\Gamma_{3-ME}$ has a maximum value 0.6602 at $\theta_{2}=1.0126$ or $\theta_{5}=2.129$. Therefore, when $\theta \in [\theta_{1},\theta_{2}]$ or $\left[\theta_{5},\theta_{6}\right]$, the entanglement order of $\Gamma_{3-ME}$ is different from 3-GM concurrence. When $\theta \in [\theta_{2},\theta_{3}]$ or $\left[\theta_{4},\theta_{5}\right]$, the entanglement order of $\Gamma_{3-ME}$ is different from 3-ME concurrence. That is, $\Gamma_{3-ME}$ is not equivalent to the 3-GM concurrence as well as the 3-ME concurrence.*

**Figure 2:**
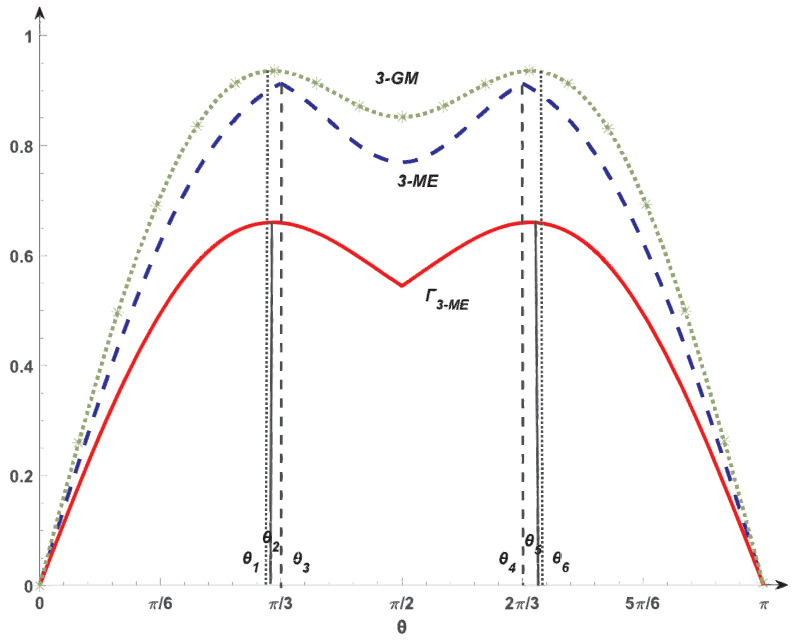
The 3-GM concurrence, 3-ME concurrence, and $\Gamma_{3-ME}$ for the four-qubit states given in (13). The dotted green, dashed blue, and solid red lines stand for the 3-GM concurrence, 3-ME concurrence, and $\Gamma_{3-ME}$ of $|\psi_{\theta }\rangle$, respectively.

Moreover, when $\theta \in (0,\theta_{2})$ or $\left(\theta_{5},\pi \right)$, we find that the 3-GM concurrence increases from 0 to $\theta_{1}$ and decreases from $\theta_{1}$ to $\theta_{2}$. Then, there always exists at least one pair of states whose 3-GM concurrences have the same value. While our measure $\Gamma_{3-ME}$ increases from 0 to $\theta_{2}$, it always has different values for $\theta \in (0,\theta_{2})$. This means that $\Gamma_{3-ME}$ is able to identify different entanglements, while the 3-GM concurrence fails in this interval. Therefore, our measure $\Gamma_{3-ME}$ is not only inequivalent to the 3-GM concurrence but also has a superior performance in characterizing the multipartite entanglement finely in this case. Similar analysis yields that our $\Gamma_{3-ME}$ distinguishes the entanglement in $\theta \in (\theta_{2},\frac{\pi }{2})$ or $\left(\frac{\pi }{2},\theta_{5}\right)$, while the 3-ME concurrence fails (Figure 2).

## 4. Conclusions

We have presented parameterized GME measures $\Gamma_{GME}$ and *k*-entanglement measures $\Gamma_{k-ME}$ in terms of *q*-concurrence. They are proved to be well-defined measures and satisfy all the related conditions such as entanglement monotonicity, invariance under local unitary transformations, convexity, and strong entanglement monotonicity. Our measures are not equivalent to the existing ones in the sense that they give rise to different state orderings. Detailed examples have shown that our measure may characterize better the genuine multipartite entanglement and the *k*-entanglement of arbitrary *n*-partite systems.

## Data Availability

Data are contained within the article.
